# Distribution Expansion of Dengue Vectors and Climate Change in India

**DOI:** 10.1029/2021GH000477

**Published:** 2022-06-01

**Authors:** Syed Shah Areeb Hussain, Ramesh C. Dhiman

**Affiliations:** ^1^ ICMR – National Institute of Malaria Research Delhi India

**Keywords:** climate change, species distribution, Aedes aegypti, Aedes albopictus, dengue

## Abstract

India has witnessed a five‐fold increase in dengue incidence in the past decade. However, the nation‐wide distribution of dengue vectors, and the impacts of climate change are not known. In this study, species distribution modeling was used to predict the baseline and future distribution of Aedine vectors in India on the basis of biologically relevant climatic indicators. Known occurrences of *Aedes aegypti* and *Aedes albopictus* were obtained from the Global Biodiversity Information Facility database and previous literature. Bio‐climatic variables were used as the potential predictors of vector distribution. After eliminating collinear and low contributing predictors, the baseline and future prevalence of *Aedes aegypti* and *Aedes albopictus* was determined, under three Representative Concentration Pathway scenarios (RCP 2.6, RCP 4.5 and RCP 8.5), using the MaxEnt species distribution model. *Aedes aegypti* was found prevalent in most parts of the southern peninsula, the eastern coastline, north eastern states and the northern plains. In contrast, *Aedes albopictus* has localized distribution along the eastern and western coastlines, north eastern states and in the lower Himalayas. Under future scenarios of climate change, *Aedes aegypti* is projected to expand into unsuitable regions of the Thar desert, whereas *Aedes albopictus* is projected to expand to the upper and trans Himalaya regions of the north. Overall, the results provide a reliable assessment of vectors prevalence in most parts of the country that can be used to guide surveillance efforts, despite minor disagreements with dengue incidence in Rajasthan and the north east, possibly due to behavioral practices and sampling efforts.

## Key Points

- Bio‐climatic factors affect the presence and abundance of the dengue vectors *Aedes aegypti* and *Aedes albopictus* in India
- Extension in the range of *Aedes aegypti* in the Thar Desert in Rajasthan is projected in view of climate change
- Range of *Aedes albopictus* is projected to extend into the upper and Trans‐ Himalayas as a result of climate change

## 1 Introduction

Dengue is the most widespread arthropod‐borne disease, that has become endemic in more than 100 countries (World Health Organization, 2020). It is usually found in tropical and sub‐tropical climates, with a vast majority of dengue cases occurring in the Americas and in South‐East Asia (World Health Organization, 2020). In India, dengue has witnessed an alarming upsurge in the past decade, with more than fivefold increase from 28,066 cases in 2010 (NVBDCP, 2010) to 157,315 cases in 2019 (NVBDCP, 2020).

The two arthropod vectors of dengue are *Aedes (Stegomyia) aegypti (L.)* and *Aedes (Stegomyia) albopictus (Skuse)*, which are also responsible for the transmission of several other arboviruses such as the chikungunya virus, yellow fever virus and Zika virus. *Aedes aegypti* exhibits an indoor resting behavior and primarily feeds on humans during the day (Scott & Takken, 2012). It is mostly found in urban areas and usually breeds in man‐made water receptacles such as plastic containers and rubber tyres (Vijayakumar et al., 2014). *Aedes albopictus* prefers to rest outdoors and is an opportunistic feeder (Paupy et al., 2009), though strong anthropophagic behavior has also been observed in some studies (Delatte et al., 2010; Ponlawat & Harrington, 2005). The presence and population size of these arthropod vectors is highly dependent on climatic factors such as temperature, rainfall and relative humidity. The poikilothermic physiology of mosquitoes renders them sensitive to temperature extremities, which affects larval development as well as vector mortality (Farjana et al., 2012). Rainfall also supports vector populations by providing suitable habitat for development of the aquatic larval stages (Farjana et al., 2012).

The drastic rise in dengue cases in India warrants a more concerted effort for dengue management and generation of suitable knowledge to support vector control. At present, no known vaccine or specific treatment for dengue exists (Gupta & Reddy, 2013). Dengue control in India is based on vector control practices such as indoor space spraying, fogging, environmental management and promotion of personal protection (NVBDCP, 2014). However, the nation‐wide distribution of dengue vectors in India is not known and the presence of aedine species has been established only in some parts of the country based on local vector surveillance such as in southern peninsular India (Selvan et al., 2016), North eastern states (Soni et al., 2018) as well as the western and eastern coastlines (Chatterjee et al., 2015; Shil et al., 2018). Moreover, climate change could significantly affect the known distribution of vectors. In recent years, Species distribution modeling (SDM) has emerged as an important tool for identifying the ecological niche and climate change induced range shifts in different species. This is particularly important for species that are vectors for pathogens and pose a human health risk. Maximum Entropy (MaxEnt v3.3.3) is a machine learning algorithm for modeling species distributions using presence‐only records. Its predictive performance is highly competitive as compared to other SDMs and has been used extensively since becoming available in 2004 (Elith et al., 2011). Therefore, in this study we used the MaxEnt model for predicting the present and future distributions of Aedine vectors of dengue in India under different climate change scenarios.

## 2 Data and Methods

### 2.1 Species Occurrence Data

Primary occurrence data for the two primary vectors of dengue in India – *Aedes aegypti* and *Aedes albopictus* were obtained from the Global Biodiversity Information Facility (GBIF ‐ https://www.gbif.org/). The records contain 562 points of occurrence of *Aedes aegypti* (GBIF.org, 2021) and 207 points of occurrence of *Aedes albopictus* (GBIF.org, 2020) in India, most of which come from a recent large‐scale study that compiled a global geographic database of *Aedes aegypti* and *Aedes albopictus* locations, derived from peer reviewed literature, national entomological surveys and expert networks (Kraemer et al., 2015). As the study included literature only up to 2014, there was a need to update the occurrence points based on new literature since 2015.

An extensive survey of all dengue entomological studies conducted in India after 2014 was carried out (Dhiman & Hussain, 2021). The search terms ‘‘India’’, ‘‘aegypti’’ and ‘‘albopictus’’ were used to find relevant peer reviewed literature in NCBI ‐ PubMed (https://www.ncbi.nlm.nih.gov/pubmed), Science Direct (https://www.sciencedirect.com/) and gray literature in Google Scholar https://scholar.google.com/. Only those studies were included where the exact coordinates of the survey were clearly mentioned. After adding these to the initial database, in total 690 occurrence points of *Aedes aegypti* and 330 occurrence points of *Aedes albopictus* were obtained. The species occurrence points were plotted in GIS environment using ArcGIS software.

### 2.2 Climatic Predictors

Climatic parameters like temperature and precipitation, are important determinants for the life cycle and survival of arthropod vectors, as well as transmission of pathogens (Farjana et al., 2012). Therefore, 19 bioclimatic variables (Table 1) that indicate the general trend, extremity and seasonality of temperature and precipitation were used as the potential predictors of vector abundance and distribution. These predictors capture information about annual and seasonal climatic conditions which are best related to species physiology, and have been used extensively for ecological niche modeling.

**Table 1:** Selected Bioclimatic Variables

Variable ID	Variable name	Selected in final model
bio 1	Annual mean temperature	No
bio 2	Mean diurnal range	Yes
bio 3	Isothermality	Yes
bio 4	Temperature seasonality	Yes
bio 5	Max. Temperature of warmest month	No
bio 6	Min. Temperature of coldest month	Yes
bio 7	Temperature annual range	No
bio 8	Mean temperature of wettest quarter	No
bio 9	Mean temperature of drienst quarter	No
bio 10	Mean temperature of warmest quarter	No
bio 11	Mean temperature of coldest quarter	No
bio 12	Annual precipitation	No
bio 13	Precipitation of wettest month	No
bio 14	Precipitation of driest month	No
bio 15	Precipitation seasonality	Yes
bio 16	Precipitation of wettest quarter	Yes
bio 17	Precipitation of driest quarter	Yes
bio 18	Precipitation of warmest quarter	Yes
bio 19	Precipitation of coldest quarter	Yes

Baseline (1970–2000) and future (2030, 2050 and 2070s) climatic data for bioclimatic variables under three RCP scenarios (RCP 2.6, RCP 4.5 and RCP 8.5), was obtained from WorldClim website (Fick & Hijmans, 2017) with a spatial resolution of 2.5 arc min (∼5 km). Future projections of climate change thus obtained, were based on the CNRM‐CM6‐1 (Voldoire et al., 2019) general circulation model developed from the Coupled Model Intercomparison Project Phase six (CMIP‐6; Eyring et al., 2016).

### 2.3 Data Processing

Data processing and modeling steps were conducted using a combination of R‐statistics (R Core Team. R, 2013), within the RStudio interface (RStudio Team, 2020), and ArcGIS® software by Esri.

Duplicate records in the species occurrence data were analyzed and removed accordingly. To account for spatial autocorrelation, spatial thinning was applied to the species occurrence records at 5 km intervals (equivalent to the resolution of environmental datasets) using the R‐package spThin (Aiello‐Lammens et al., 2015). The final species occurrence data contained 383 and 205 spatially explicit records of *Aedes aegypti* and *Aedes albopictus* respectively. The species occurrence records, were used to construct a sampling bias layer in order to account for differences in sampling efforts across different locations.

In order to reduce model complexity, highly collinear variables that did not contribute significantly to the model output were eliminated. A cross‐correlation table (Table S1 in Supporting Information S1) was used to identify variables that show strong collinearity (>0.8), and a cluster dendrogram of variables grouped based on collinearity was constructed (Figure S1 in Supporting Information S1). Initial models were run using all bioclimatic variables, and the contribution of each variable to model output was determined. Variables with low contribution to model outputs and strong collinearity (>0.8) with other variables were eliminated one by one in subsequent models to obtain the final list of non‐collinear bioclimatic variables. At each stage, the effect of eliminating a variable on model performance was assessed based on the AUC value ‐ area under the ROC (Receiver operating characteristic) curve. The selected variables were finally reviewed and approved through expert opinion (Table 1).

### 2.4 Predictive Modeling

Present and future distribution of *Aedes aegypti* and *Aedes albopictus* was evaluated using Maxent (v 3.4.1; Philips et al., 2004) with the help of the R package ENMTML (Andrade et al., 2020). Maxent is a presence‐only species distribution model that employs a machine learning algorithm to generate a probability distribution of the selected species, and has been shown to be effective even with low number of sampling points (Townsend Peterson et al., 2007). The Maxent model relies on Baye's rule (Equation 1) to estimate the probability density of the species distribution in covariate space, by maximizing the entropy/dispersion across the geographic space (Elith et al., 2011).

(1) $$P\left(y=1|x\right)=\frac{P\left(x|y=1\right)P\left(y=1\right)}{P\left(x\right)}$$

where, y denotes the presence (*y* = 1) or absence of the species (*y* = 0). P(*x* = 1|y) = π(*x*) is the probability density of covariates across the presence locations of species. P(*y* = 1|x) is the probability of presence of species, given the covariate density. P(*y* = 1) is the prevalence of the species. P(*x*) = 1/|x| is the probability density of the covariates. As Maxent relies on presence records only, P (*y* = 1|x) cannot be determined directly, and hence an estimation of the distribution of π(*x*) is made (Philips et al., 2004). The Maxent distribution is a Gibbs distribution derived from a set of features f_i_, with feature weights λ_i_, and is defined by the equation

(2) $$q_{\lambda }\left(x\right)=\frac{\exp\left(\sum_{i=1}^{n}\lambda_{i}f_{i}\left(x\right)\right)}{Z_{\lambda }}$$

where Z_λ_ is the normalization constant. In order to estimate this distribution, Maxent employs the principle of maximum entropy to Shannon's information theory based on the equation

(3) $$H=q_{\lambda }\left(x\right)\text{ln}\;q_{\lambda }\left(x\right)$$

where H is the maximum entropy of the system.

Model parameters were determined by hit and try method, wherein initial models were run with five levels of complexity (linear, linear‐quadratic, hinge, linear‐quadratic‐hinge and linear‐quadratic‐hinge‐polynomial) and 20 regularization multipliers from 1 to 10 with a half step interval in between. The outputs were analyzed based on the omission rate with respect to the testing data, Akaike Information Criterion score and AUC values. Based on these, the best set of parameters for the maxent model was selected. Pseudo absences were allocated randomly after applying appropriate environmental and geographical constraints (50 km buffer). For validation of model outputs, k‐fold cross validation was used to partition the presence data into five subsets. The outputs were obtained in the form of GeoTiff rasters containing the logistic suitability score as the values of the pixels for the baseline and each of the future projections.

The continuous logistic outputs were then converted to binary outputs using the ‘‘maximum test for sensitivity and specificity (MAXTSS)’’ in MaxEnt, which has been identified as the best method for threshold selection in presence only models (Liu et al., 2005). The results were plotted in ArcGIS to assess the risk of range expansion in the vectors.

### 2.5 Validation of Model Outputs

A number of different evaluation metrics were used for assessing the model performance. The traditional accuracy measures (AUC and Kappa/True Skill Statistic ‐ TSS) have often been criticized due to their over‐dependence on species prevalence and can give misleadingly high values by not penalizing over prediction (Allouche et al., 2006). Therefore, similarity indices – namely Jaccard and Sorensen, which are not biased by true negatives were also evaluated. Most evaluation metrics are constructed for presence‐absence models and modified accordingly for presence‐only models. Therefore, to ensure model reliability, the Boyce index which is specifically a presence‐only metric, was also computed. The significance of selected bioclimatic variables in model outputs was assessed by permutation importance contribution.

## 3 Results

### 3.1 Variables' Contribution and Selection

The cross‐correlation table and cluster dendrogram revealed groups of variables which showed very high collinearity. Low contributing and collinear variables were eliminated one by one, after running multiple preliminary models. The final list of variables with low collinearity and significant contribution to outputs is presented in Table 1.

### 3.2 Evaluation of Model Performance

Three types of evaluation metrics were computed for *Aedes aegypti* and *Aedes albopictus* model outputs (Table 2) – accuracy metrics (AUC and TSS), similarity indices (Jaccard and Sorensen) and reliability metrics (Continuous Boyce Index).

**Table 2:** Accuracy and Reliability Metrics for the Validation of Model Outputs

Variable	*Aedes aegypti*		*Aedes albopictus*	
	Coefficient	Sd	Coefficient	Sd
AUC	0.94	0.01	0.95	0.04
TSS	0.77	0.04	0.84	0.11
Jaccard	0.80	0.03	0.85	0.09
Sorensen	0.89	0.02	0.92	0.05
OR	0.06	0.03	0.07	0.06
Boyce	0.86	0.03	0.84	0.08

The AUC values for both *Aedes aegypti* and *Aedes albopictus* were significantly high (0.94 and 0.95 respectively) indicating strong agreement between the training and testing datasets. The threshold dependent TSS values were also significantly high for the two species (0.77 and 0.84) indicating that model performance was very good. Similarity indices such as Jaccard and Sorensen were identified as an alternative to the traditional accuracy metrics that measure the similarity between the model outputs and validation datasets. Significantly high values of the Jaccard (0.80 and 0.85) and Sorensen indices (0.89 and 0.92) for both the vectors also indicate that the model was able to accurately predict vector prevalence. Similarly, high values of Boyce index (0.86 and 0.84) for the model outputs indicates that model performance was excellent.

**Figure 1:**
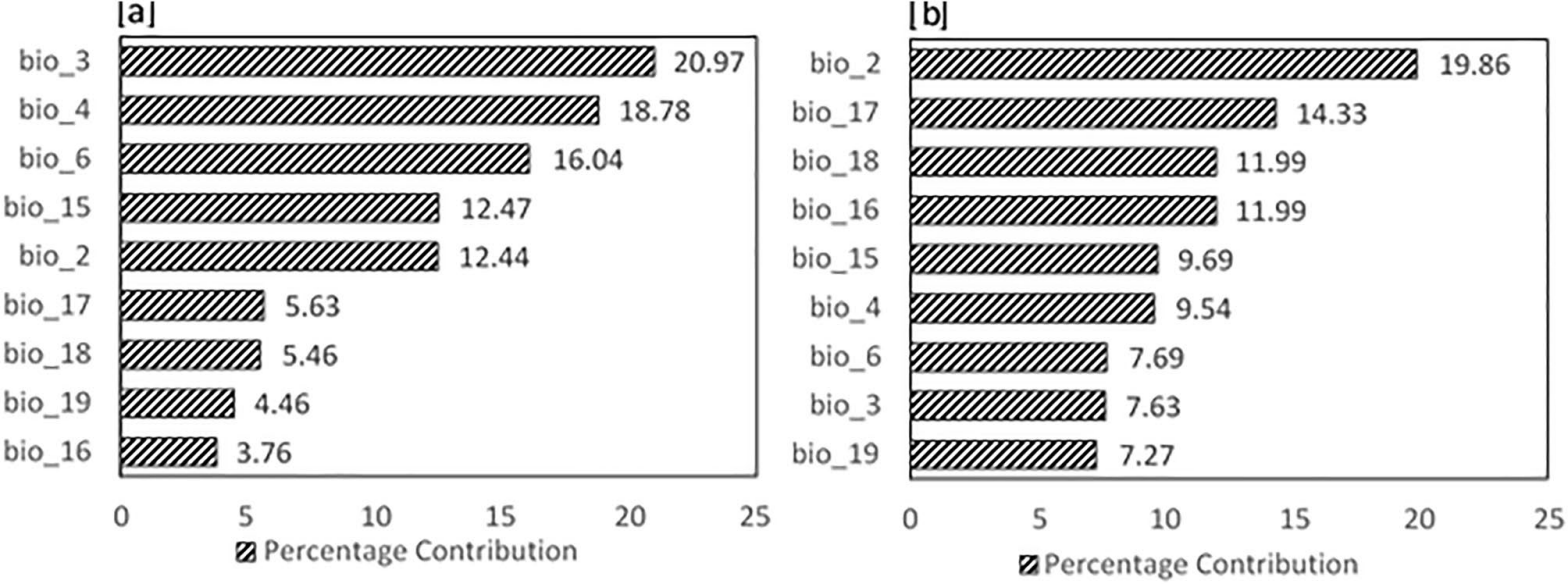
Variable Contributions to model outputs for (a) Aedes aegypti and (b) Aedes albopictus.

The variables which contributed most to model outputs (Figure 1) for *Aedes aegypti* were found to be the isothermality (bio3), temperature seasonality (bio4) and the minimum temperature of the coldest month (bio6). On the other hand, for the prevalence of *Aedes albopictus* mean diurnal range (bio2), precipitation of the driest quarter (bio17) and precipitation of the warmest quarter (bio18) were found as important variables. This indicates that temperature may be an important limiting factor for *Aedes aegypti*, whereas precipitation is the limiting factor for *Aedes albopictus*.

### 3.3 Baseline and Projected Future Distribution of *Aedes aegypti* and *Aedes albopictus*

**Figure 2:**
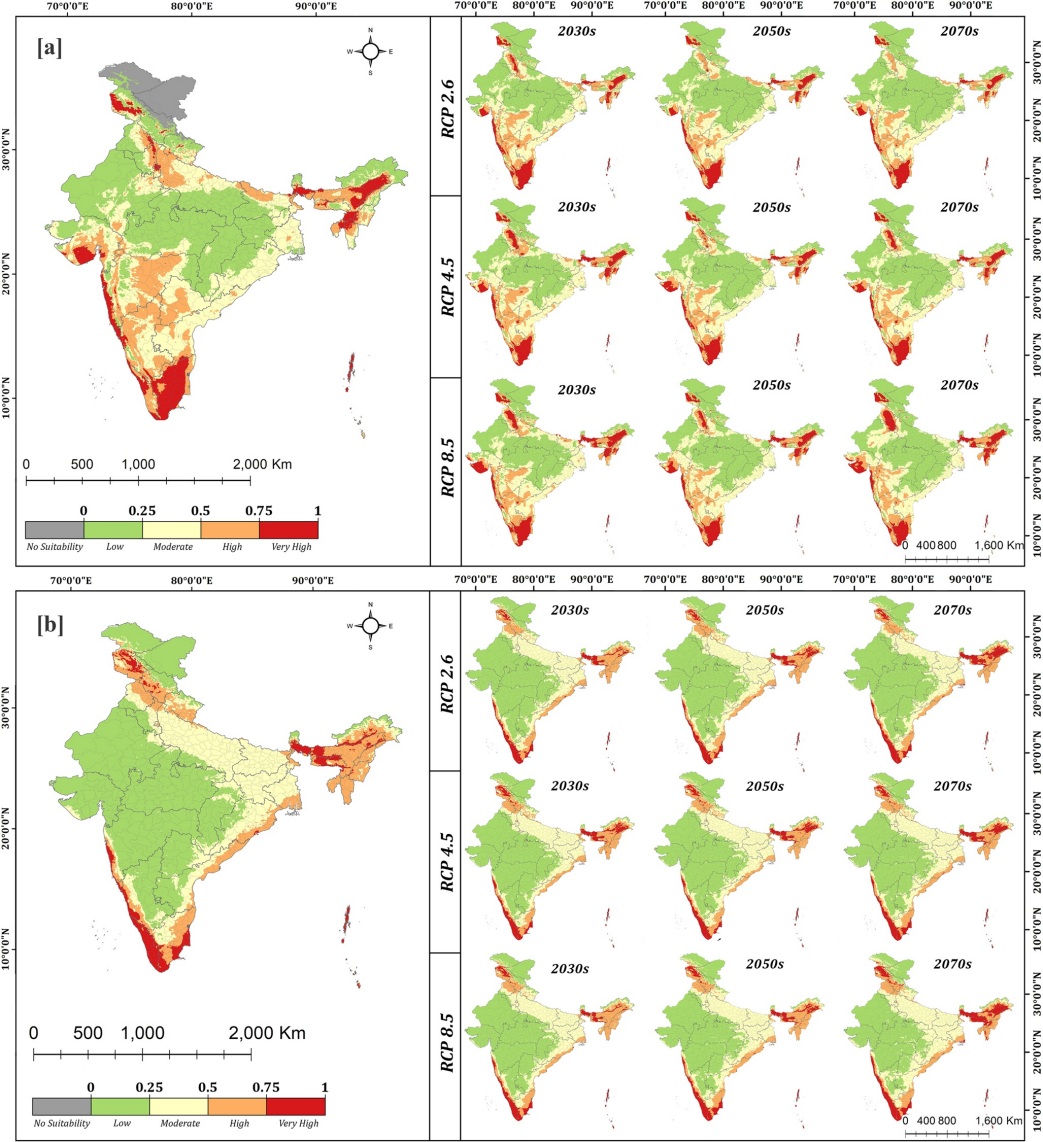
Baseline and projected future suitability of (a) *Aedes aegypti* and (b) *Aedes albopictus* under different climate change scenarios, based on the nine selected bio‐climatic variables, using MaxEnt species distribution modeling. Local changes in the distribution of *Aedes aegypti* are visible in Gujarat, Haryana, Punjab, north east and the southern peninsular plateau. In contrast, *Aedes albopictus* witnesses local variations in distribution in north east and the Himalayan regions.

Based on the probability distribution maps generated from maxent logistic output (Figure 2), the baseline distribution of *Aedes aegypti* was found very high in the Kashmir valley (0.63–0.91), Malwa plains of Punjab (0.59–0.76) and Haryana (0.65–0.88), Saurashtra region of Gujarat (0.4–0.79), upper Brahmaputra and Barak valley in Assam (0.69–0.88), the Konkan coastline (0.75–0.95) and the southern peninsular plains (0.61–0.96). The vector had high focal prevalence in the urbanized western regions of Uttar Pradesh (UP; 0.51–0.65), Delhi (0.76–0.88), some northern districts of Bihar (0.48–0.67) and the northern Jalpaiguri division of West Bengal (0.56–0.93).

**Figure 3:**
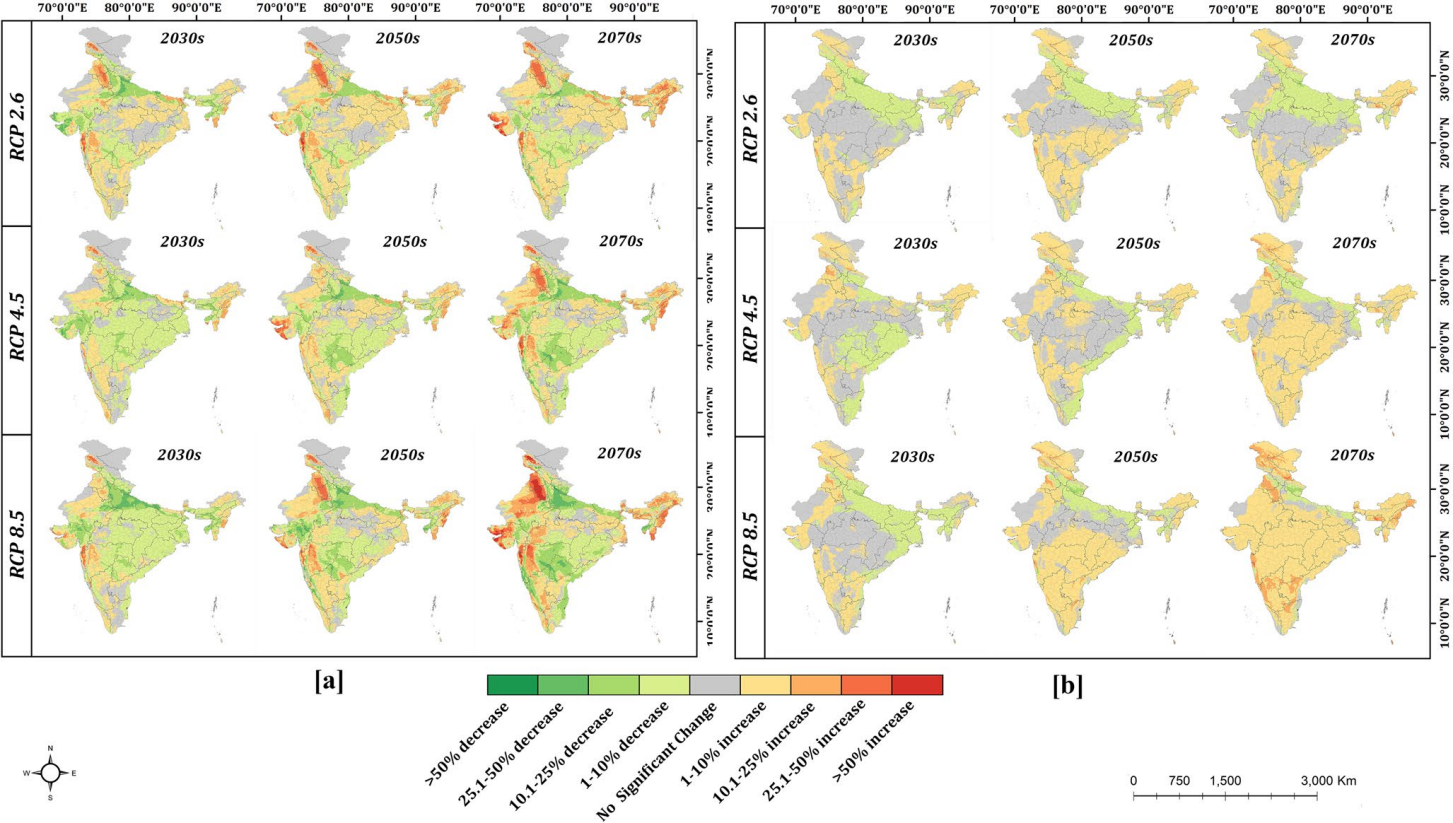
Change in suitability for (a) *Aedes aegypti* and (b) *Aedes albopictus* in future scenarios of climate change as compared to the baseline suitability. While *Aedes aegypti* is projected to witness significant changes in many parts of the country, substantial changes in distribution of *Aedes albopictus* are mostly limited to a few regions in the north east and Jammu & Kashmir.

A few regions of the Deccan plateau and northern Indo‐Gangetic plains also had moderate to high (0.25–0.75) distribution of *Aedes aegypti*. Most of the central highlands, the Thar Desert region and the greater Himalayan regions of Jammu & Kashmir have very low prevalence (>0.25) of *Aedes aegypti*. The vector is found absent in the trans‐Himalayan regions of Jammu & Kashmir and Ladakh (Figure 2a).

The prevalence of *Aedes albopictus* was found very high along the Coromandel (0.63–0.98), Malabar (0.88–0.97), and Konkan coastline (0.62–0.81), southern Western Ghats (0.79–0.99), Kashmir valley (0.68–0.85), lower Brahmaputra valley, Kamrup and Goalpara hills in Assam (0.71–0.8) as well as the Himalayan and terai regions of West Bengal (0.74–0.89). In the north eastern region, both vectors are prevalent but, *Aedes albopictus* appears to be the dominant vector with more widespread distribution (Figure 2b). For example, in Arunachal Pradesh, *Aedes albopictus* was significantly more abundant than *Aedes aegypti*, which is restricted only to the lesser Himalayas. In the Indo‐Gangetic plains and Eastern Ghats (0.28–0.54), *Aedes albopictus* had mostly moderate (0.29–0.49) prevalence in the baseline years, whereas a large part of India, that is, arid/semi‐arid regions of Rajasthan, Gujarat, most parts of Deccan plateau and the central highlands show low prevalence (0.04–0.18) of *Aedes albopictus*. Future projections of climate change were based on three scenarios – the low emissions scenario (RCP 2.6), moderate emissions scenario (RCP 4.6) and high emissions scenario (RCP 8.5). The RCP 2.6 scenario of climate change projects a twofold increase in geographic area with very high prevalence of *Aedes aegypti* in Punjab and Haryana, and a further 18.3% increase in area by 2070s. However, an initial reduction in suitability of *Aedes aegypti* is projected in the Saurashtra and Kachchh regions of Gujarat (12%–32%), Jalpaiguri division of West Bengal (5%–9%) and north eastern states (10%–16%) by 2030s. This is followed by a substantial increase in suitability by 2050 and 2070s in Gujarat (9%–34% and 10%–40%) and in the Barak valley region of the north east (10%–21% and 10%–24%) (Figure 3). Some reduction in suitability is also observed in the Rohilkhand and Awadh plains of Uttar Pradesh (10%–28% in 2030s, 10%–19% in 2050s and 11%–24% in 2070s). The RCP 4.5 scenario projects a significant reduction in suitability for *Aedes aegypti* by 2030s in Haryana (10%–15%), Punjab (3%–13%), Delhi (9%–15%), Rohilkhand and Awadh plains of Uttar Pradesh (10%–26%), Saurashtra regions of Gujarat (11%–21%), Tripura (14%–16%), Meghalaya (11%–16%) and the upper Brahmaputra valley of Assam (7%–13%). The suitability for *Aedes aegypti* reduces further in western UP (11%–26% in 2050s, 11%–28% in 2070s), but increases considerably in Gujarat by 2050s (15%–34%) as well as in Punjab (13%–31%) and Haryana (10%–31%) by 2070s. Similarly, under RCP 8.5, a significant reduction in suitability for *Aedes aegypti* is projected in Punjab, Haryana, the Indo‐Gangetic plains, most of Gujarat, north east and eastern regions as well as in the southern peninsular plateau by 2030s. The reduction in suitability continues in 2050 and 2070s in the southern peninsular plateau, with a 13.4% contraction in very high suitability areas by 2070s. However, the suitability for *Aedes aegypti* increases considerably in 2050 and 2070s in Punjab (12%–60%), Haryana (22%–65%), Gujarat (10%–40%), Meghalaya (10%–24%) and Mizoram (17%–36%). In Nagaland and the Konkan coast of Maharashtra, suitability for *Aedes aegypti* increases under all future years, with most significant rise in 2070s (13%–31% and 15%–32% respectively). Furthermore, *Aedes aegypti* is projected to invade several regions of Leh (Ladakh) and northern Himachal Pradesh which are unsuitable for *Aedes aegypti* in baseline years. Increase in the suitability for *Aedes aegypti* in Punjab, Haryana, Gujarat and the North East under most future scenarios may be attributable to the decline in DTR ‐ Diurnal Temperature Range (bio 2), based on the results from the model. Earlier research has also highlighted the detrimental role of high daily temperature fluctuations on vector survival, which is the most likely cause for increased suitability (Lambrechts et al., 2011). Reduced suitability in the Central Highlands and the southern peninsular plateau under future years may be linked with decrease in the minimum temperature of the coldest month (bio 6), which coupled with notable increase in temperature seasonality (bio 4) is likely to promote seasonal prevalence of *Aedes aegypti* in this region.

The suitability for *Aedes albopictus* is not expected to change substantially in the country, though some local changes in suitability are visible from the logistic distribution and change maps (Figure 3). Under RCP 2.6, the suitability for *Aedes albopictus* increases gradually in the upper Brahmaputra valley of Assam, with as much as 40% and 122% increase in geographic area of very high suitability in the 2050 and 2070s respectively. Minor reduction in suitability is also observed in the terai regions of Uttarakhand (5%–12%). Similar changes are projected in RCP 4.5. However, under RCP 8.5 significant increase in suitability is projected in Meghalaya and lower Brahmaputra valley (11%–19%), in addition to the upper Brahmaputra valley. Suitability for *Aedes albopictus* does not change significantly in future years in the semi‐arid and arid regions and the central highlands under all three scenarios of climate change. Reduced suitability in terai region of Uttarakhand under future years is likely due to a decline in rainfall in the region under most climate change scenarios, projected in the precipitation of wettest quarter (bio 16), precipitation of driest quarter (bio 17) and the precipitation of the warmest quarter (bio 18) variables. On the other hand, increasing precipitation of the warmest quarter (bio 18) in the north east under all future scenarios is associated with an increase in suitability for *Aedes albopictus*. Unlike *Aedes aegypti*, which has adapted to urban environments and can grow in household containers, *Aedes albopictus* is more dependent on water availability, and is therefore sensitive to changes in precipitation under future scenarios (Mogi et al., 2015).

### 3.4 Projected Range Expansion of Vectors

**Figure 4:**
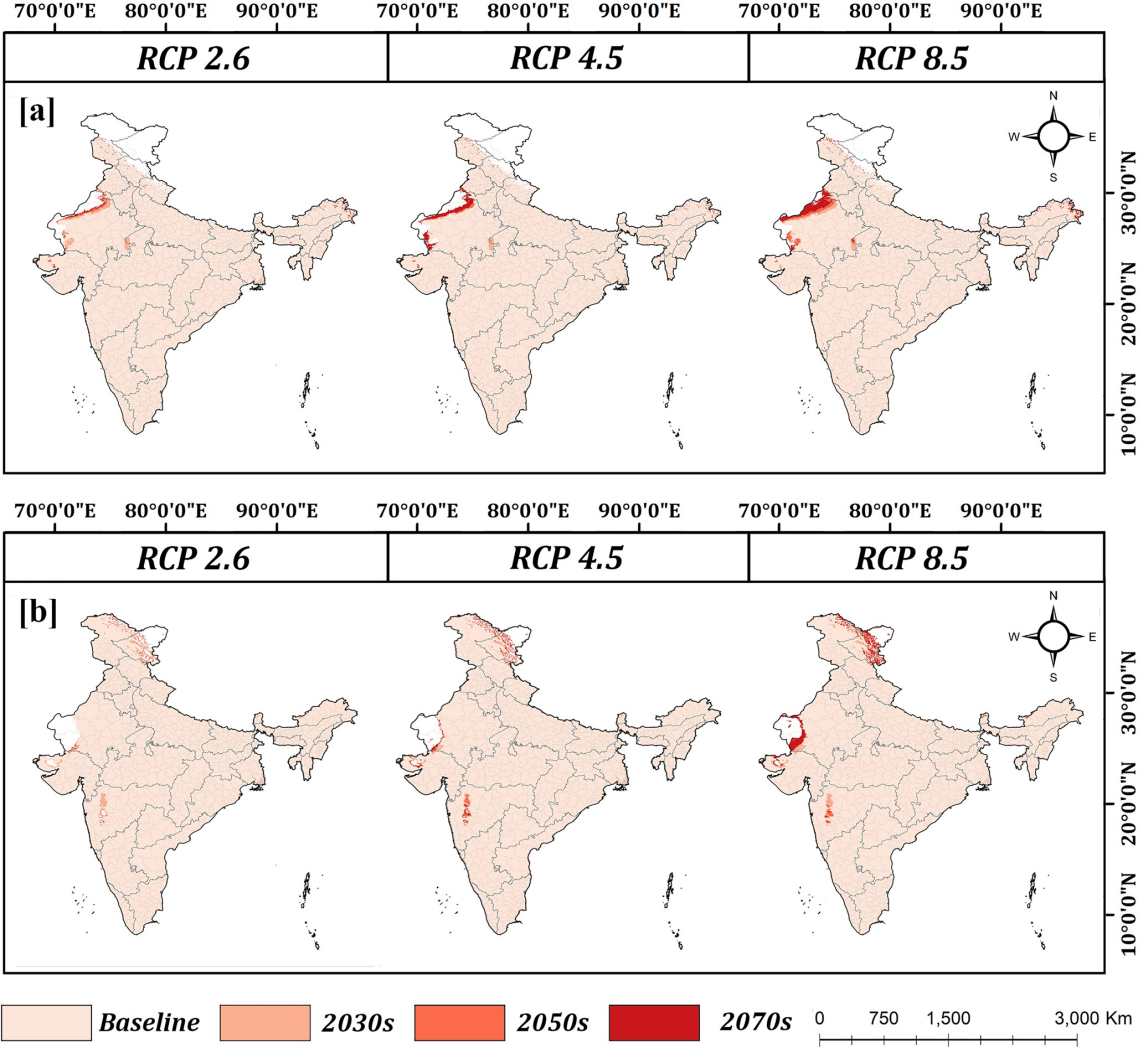
Projected range expansion of (a) *Aedes aegypti* and (b) *Aedes albopictus* in future years under different climate change scenarios using the maximum of sensitivity and specificity as the threshold values for vector range.

The binary outputs generated by using the MaxTSS as the presence threshold (Figure 4), project an expansion in the distribution of *Aedes aegypti* at the edges of the Thar Desert in Rajasthan, by 2030, 2050 and 2070s. This expansion is most prominent in the RCP 8.5 scenario, and by 2070s, almost all of Rajasthan is projected to be suitable for *Aedes aegypti*. Earlier studies have also observed the persistence of *Aedes aegypti* in arid urban environments (Kaul & Rastogi, 1997; Marinho et al., 2016). Their close association with human habitats, tendency to breed in small containers and ability of eggs to withstand dessication have been theorized as the possible causes for this (Coalson et al., 2018; Reinhold et al., 2018). Minor increase in range of *Aedes aegypti* is also projected in the upper Himalayas of Arunachal Pradesh.

On the other hand, the results project a substantial expansion of *Aedes albopictus* in the Leh (Ladakh) regions comprising of the upper and trans‐Himalayas (Figure 4). *Aedes albopictus* has been established as a cold adapted species (Reinhold et al., 2018). Under present conditions it is already predicted to have a sizable population in the lesser Himalayan region of Jammu and Kashmir. Climate change is projected to increase temperatures by approximately 1.5–2°C by 2030s, 2.75–3.2°C in 2050s and 2.15–5°C in 2070s in the Himalayan region under different climate cange scenarios (based on data used for the study), which is likely to accelerate the developmental cycle of Aedes mosquitoes. Significant increase in range of *Aedes albopictus* is also projected in the Jaisalmer district of Rajasthan.

## 4 Discussion and Conclusions

In India, several studies have been undertaken on the projected scenario of malaria and dengue with respect to climate change (Dhiman et al., 2011; Sarkar et al., 2019), while there are negligible studies on the altered distribution of vectors (Kraemer et al., 2019; Ogden et al., 2014). Furthermore, the alarming rise in dengue in the last decade has received relatively less attention (Gupta & Reddy, 2013). The present study has found widespread distribution of dengue vectors in India, with a significant risk of expansion in some parts of Thar Desert and upper Himalayas, due to climate change. In north east India as well as the western coastline, both *Aedes aegypti* and *Aedes albopictus* have high prevalence, which implies that the risk of dengue is high, though the reported cases of dengue do not reflect this (NVBDCP, 2020). Such areas warrant constant monitoring and increased surveillance for dengue incidence. *Aedes aegypti* was found more prevalent in the Deccan plateau and the semi‐arid regions of Gujarat and Rajasthan, while *Aedes albopictus* in the eastern coastline.

*Aedes aegypti* is projected to witness more widespread increase in distribution under RCP 2.6 in 2030 and 2050s, whereas marginal reduction is observed in most parts of the country under RCP 4.5 and 8.5. By 2070s, RCP 8.5 demonstrates a significant increase in suitability for *Aedes aegypti* in the eastern parts of the country. In contrast, the suitability for *Aedes albopictus* remains largely similar in most parts of the country by 2030s. Increase in the abundance of *Aedes albopictus* is projected in southern India, upper Himalayan regions of Leh (Ladakh) and Arunachal Pradesh by 2050s under RCP 8.5, and by 2070s. *Aedes albopictus* has been identified as a cold‐adapted species in earlier studies (Tippelt et al., 2020).

The states which regularly report high incidence of dengue, namely Gujarat, Maharashtra, Punjab and Karnataka (NVBDCP, 2020) are also predicted to have very high distribution of *Aedes aegypti* and/or *Aedes albopictus*. On the other hand, the model outputs are in disagreement with dengue incidence in the states of Rajasthan and north‐eastern parts (NVBDCP, 2020). In Rajasthan, the distribution of both the vectors is low but the incidence of dengue is high that is, Rajasthan ranked four in dengue incidence in the country in 2019 (NVBDCP, 2020). A study undertaken in 1997 (Kaul & Rastogi, 1997) found perennial prevalence of *Aedes aegypti* in Rajasthan (Kaul & Rastogi, 1997) which could not be captured by our models. The water storage practices in dry parts of Rajasthan were perhaps not captured by the climatic variables suitable for Aedes. In North eastern states, it is just the opposite, which can be explained by oversampling efforts in the north eastern states (NVBDCP, 2020). Further studies are warranted to ascertain the reasons for low incidence in north eastern states as well as the future risk of dengue in view of climate change.

A striking observation in our study was that temperature related factors (bio3, bio4, bio6) contributed more significantly to the suitability of *Aedes aegypti*, whereas precipitation related factors (bio16, bio17, bio18) contributed more significantly to the suitability of *Aedes albopictus*. This difference is most likely a result of the differences in habitat preference of the two species. As discussed previously, breeding of *Aedes aegypti* in household containers enables it to breed in low precipitation conditions due to water storage practices of the community. At the same time, *Aedes albopictus* has a larger temperature tolerance (Tippelt et al., 2020), due to which precipitation is a more significant limiting factor for *Aedes albopictus*.

Our study provides insights on baseline as well as projected distribution of Aedes aegypti and *Aedes albopictus* in India. The models are based on the assumption that there are no other dispersal limitations for the two vectors, therefore, may not represent the real scenario as the actual realized niche of the species may differ based on local factors (such as the water storage practices) which cannot be captured by country‐wide models. Moreover, variability in resolution of sampling can introduce bias to model results, as observed in the north east.

The areas with projected expansion in range warrant strengthened efforts for entomological as well as dengue surveillance. The projected maps thus generated may be useful in guiding the ground surveillance efforts in projected areas of distribution of both the vectors.

## Conflict of Interest

The authors declare no conflicts of interest relevant to this study.

## Supporting information

Supporting Information S1

## Data Availability

Primary occurrence locations of Aedine vectors in India was obtained from the GBIF database (https://www.gbif.org/). The GBIF occurrences data set used for *Aedes aegypti* is available at (https://doi.org/10.15468/dl.b63mgt) and that for *Aedes albopictus* is available at (https://doi.org/10.15468/dl.jub5cx). The occurrence datasets include data from a large scale study that compiled occurrence coordinates from literature upto 2014 (Kraemer et al., 2015). An extensive literature survey was conducted to find Aedes occurrences in literature published after 2014. The data of these occurrences has been published in the dryad data repository (Dhiman & Hussain, 2021) and is available from the doi: https://doi.org/10.5061/dryad.6wwpzgmzq. Data for baseline and projected (RCP 2.6, RCP 4.5 and RCP 8.5) bioclimatic variables was obtained from WorldClim (Fick & Hijmans, 2017) at 2.5 arc min resolution. Future projections of climate change thus obtained, were based on the CNRM‐CM6‐1 (Voldoire et al., 2019) general circulation model developed from the Coupled Model Intercomparison Project Phase six (CMIP‐6; Eyring et al., 2016).
