# Supplementary material for: Distribution Expansion of Dengue Vectors and Climate Change in India
Source: Geohealth. 2022 Jun 1;6(6):e2021GH000477. doi: 10.1029/2021GH000477 (PMC9210256; doi:10.1029/2021GH000477)
Supplement: Supplementary file 1 — Supporting Information S1 [file GH2-6-e2021GH000477-s001.docx]

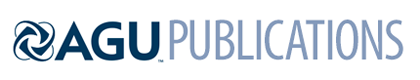


*GeoHealth*

Supporting Information for

**Distribution Expansion of Dengue vectors and Climate Change in India**

Syed Shah Areeb Hussain ^1^, Ramesh C. Dhiman ^1*^

^1^ ICMR- National Institute of Malaria Research

* **Corresponding Author:** Dr. R. C. Dhiman (r.c.dhiman@gmail.com)

**Contents of this file**

Figures S1 to S2

Tables S1

**Introduction**

The figures and graphs presented as supplementary files provide additional information to support the methodology, results and conclusions of the study. This includes the dataset and variables used in the study as well as additional tables and figures that provide greater insight into the methodology employed for modelling vector distributions and the results obtained.

Figure S1 and Table S1 provide greater detail of the multi-collinearity test that was used for the selection of variables. Figure S2 represents the responses of the selected variables at the occurrence locations.


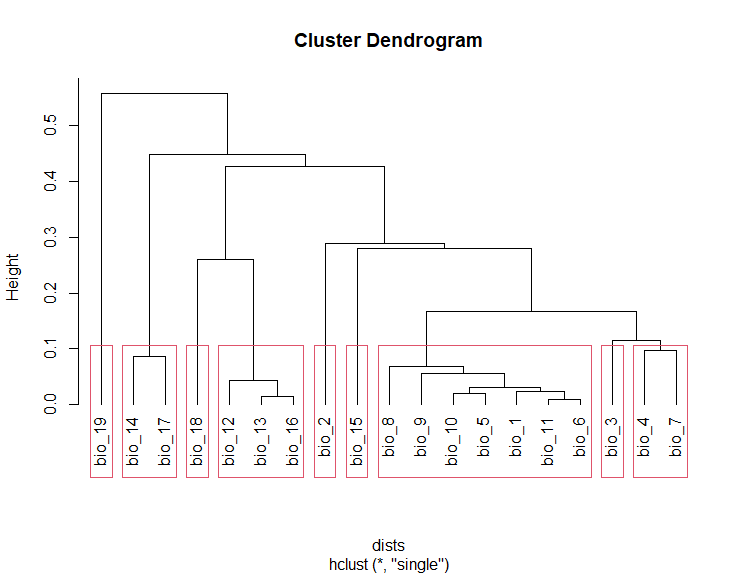


Figure S1. Cluster dendrogram of variables grouped based on results of the multi-collinearity test.

***
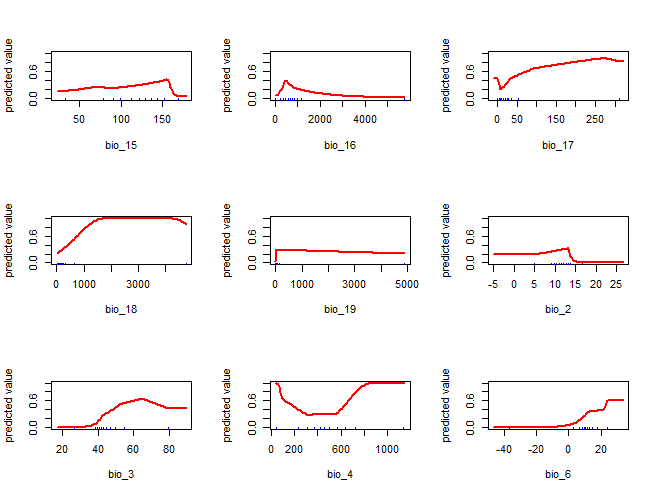
***

Figure S2. Variable response curves for the selected bioclimatic variables.

|  | bio1 | bio2 | bio3 | bio4 | bio5 | bio6 | bio7 | bio8 | bio9 | bio10 | bio11 | bio12 | bio13 | bio14 | bio15 | bio16 | bio17 | bio18 | bio19 |
| --- | --- | --- | --- | --- | --- | --- | --- | --- | --- | --- | --- | --- | --- | --- | --- | --- | --- | --- | --- |
| bio1 | 1.00 | 0.17 | 0.56 | -0.63 | 0.92 | 0.95 | -0.35 | 0.93 | 0.94 | 0.97 | 0.98 | 0.20 | 0.24 | -0.24 | 0.51 | 0.25 | -0.23 | -0.03 | 0.01 |
| bio2 | 0.17 | 1.00 | -0.18 | 0.41 | 0.47 | -0.09 | 0.73 | 0.26 | 0.17 | 0.33 | 0.03 | -0.57 | -0.38 | -0.44 | 0.57 | -0.42 | -0.47 | -0.43 | -0.29 |
| bio3 | 0.56 | -0.18 | 1.00 | -0.87 | 0.31 | 0.72 | -0.77 | 0.42 | 0.56 | 0.39 | 0.69 | 0.27 | 0.26 | -0.03 | 0.01 | 0.22 | -0.12 | 0.02 | 0.28 |
| bio4 | -0.63 | 0.41 | -0.87 | 1.00 | -0.34 | -0.73 | 0.90 | -0.47 | -0.61 | -0.45 | -0.78 | -0.46 | -0.41 | 0.02 | -0.03 | -0.42 | 0.06 | -0.14 | -0.21 |
| bio5 | 0.92 | 0.47 | 0.31 | -0.34 | 1.00 | 0.78 | 0.01 | 0.88 | 0.88 | 0.98 | 0.84 | -0.04 | 0.08 | -0.36 | 0.65 | 0.08 | -0.35 | -0.24 | -0.10 |
| bio6 | 0.95 | -0.09 | 0.72 | -0.73 | 0.78 | 1.00 | -0.62 | 0.83 | 0.90 | 0.86 | 0.99 | 0.33 | 0.33 | -0.14 | 0.33 | 0.34 | -0.15 | 0.03 | 0.11 |
| bio7 | -0.35 | 0.73 | -0.77 | 0.90 | 0.01 | -0.62 | 1.00 | -0.22 | -0.33 | -0.14 | -0.52 | -0.58 | -0.44 | -0.23 | 0.30 | -0.45 | -0.21 | -0.35 | -0.31 |
| bio8 | 0.93 | 0.26 | 0.42 | -0.47 | 0.88 | 0.83 | -0.22 | 1.00 | 0.84 | 0.93 | 0.87 | 0.16 | 0.20 | -0.27 | 0.59 | 0.22 | -0.24 | 0.04 | -0.05 |
| bio9 | 0.94 | 0.17 | 0.56 | -0.61 | 0.88 | 0.90 | -0.33 | 0.84 | 1.00 | 0.92 | 0.93 | 0.16 | 0.24 | -0.21 | 0.49 | 0.23 | -0.22 | -0.11 | 0.05 |
| bio10 | 0.97 | 0.33 | 0.39 | -0.45 | 0.98 | 0.86 | -0.14 | 0.93 | 0.92 | 1.00 | 0.91 | 0.08 | 0.15 | -0.29 | 0.59 | 0.16 | -0.28 | -0.12 | -0.05 |
| bio11 | 0.98 | 0.03 | 0.69 | -0.78 | 0.84 | 0.99 | -0.52 | 0.87 | 0.93 | 0.91 | 1.00 | 0.28 | 0.31 | -0.20 | 0.42 | 0.31 | -0.21 | 0.00 | 0.06 |
| bio12 | 0.20 | -0.57 | 0.27 | -0.46 | -0.04 | 0.33 | -0.58 | 0.16 | 0.16 | 0.08 | 0.28 | 1.00 | 0.92 | 0.26 | -0.02 | 0.96 | 0.34 | 0.70 | 0.41 |
| bio13 | 0.24 | -0.38 | 0.26 | -0.41 | 0.08 | 0.33 | -0.44 | 0.20 | 0.24 | 0.15 | 0.31 | 0.92 | 1.00 | 0.04 | 0.25 | 0.99 | 0.11 | 0.48 | 0.42 |
| bio14 | -0.24 | -0.44 | -0.03 | 0.02 | -0.36 | -0.14 | -0.23 | -0.27 | -0.21 | -0.29 | -0.20 | 0.26 | 0.04 | 1.00 | -0.54 | 0.08 | 0.92 | 0.35 | 0.18 |
| bio15 | 0.51 | 0.57 | 0.01 | -0.03 | 0.65 | 0.33 | 0.30 | 0.59 | 0.49 | 0.59 | 0.42 | -0.02 | 0.25 | -0.54 | 1.00 | 0.21 | -0.52 | -0.22 | -0.11 |
| bio16 | 0.25 | -0.42 | 0.22 | -0.42 | 0.08 | 0.34 | -0.45 | 0.22 | 0.23 | 0.16 | 0.31 | 0.96 | 0.99 | 0.08 | 0.21 | 1.00 | 0.16 | 0.55 | 0.41 |
| bio17 | -0.23 | -0.47 | -0.12 | 0.06 | -0.35 | -0.15 | -0.21 | -0.24 | -0.22 | -0.28 | -0.21 | 0.34 | 0.11 | 0.92 | -0.52 | 0.16 | 1.00 | 0.43 | 0.19 |
| bio18 | -0.03 | -0.43 | 0.02 | -0.14 | -0.24 | 0.03 | -0.35 | 0.04 | -0.11 | -0.12 | 0.00 | 0.70 | 0.48 | 0.35 | -0.22 | 0.55 | 0.43 | 1.00 | 0.04 |
| bio19 | 0.01 | -0.29 | 0.28 | -0.21 | -0.10 | 0.11 | -0.31 | -0.05 | 0.05 | -0.05 | 0.06 | 0.41 | 0.42 | 0.18 | -0.11 | 0.41 | 0.19 | 0.04 | 1.00 |

Numbers marked in red indicate high collinearity

Table S1. Pearson’s correlation for testing multi-collinearity between bioclimatic variables.
